# Solid Pseudopapillary Neoplasm of the Pancreas in an Adolescent: An Uncommon Finding

**DOI:** 10.7759/cureus.102540

**Published:** 2026-01-29

**Authors:** Ana Sofia Nunes, Sofia Martinho, Luciana Abelha, Carlos Veiga, Andreia Felizes, Vera Gonçalves

**Affiliations:** 1 Department of Paediatrics, Unidade Local de Saúde de Braga, Braga, PRT; 2 Department of Paediatric Surgery, Unidade Local de Saúde de Braga, Braga, PRT; 3 Department of Paediatrics, Unidade Local de Saúde do Alto Minho, Viana do Castelo, PRT; 4 Department of General Surgery, Unidade Local de Saúde de Braga, Braga, PRT

**Keywords:** child and adolescent, paediatric radiology, paediatric surgery, pancreatic tumors, solid pseudopapillary neoplasm

## Abstract

Solid pseudopapillary neoplasm (SPN) of the pancreas is a rare tumor in children with low malignant potential. It predominantly affects adolescent females and is frequently diagnosed incidentally. We report the case of an 11-year-old girl presenting with acute abdominal pain, fever, vomiting, and anorexia. Physical examination and initial ultrasound were inconclusive. Cross-sectional imaging via computed tomography (CT) and magnetic resonance imaging (MRI) revealed a large, heterogeneous mass in the pancreatic tail. Laboratory evaluation, including tumor markers, was unremarkable. She was managed initially with antibiotics for suspected inflammatory complications. After stabilization, she underwent laparoscopic spleen-preserving distal pancreatectomy. Immunohistochemical staining was positive for beta (β)-catenin, CD56, cyclin D1, and synaptophysin, and negative for chromogranin. No regional lymph node metastases were found (pT3N0M0).

SPN, though rare in children, should be considered in the differential diagnosis of pancreatic masses. Surgical resection offers an excellent prognosis. Immunohistochemistry, particularly β-catenin staining, is essential for diagnosis.

## Introduction

In the pediatric population, pancreatic masses are rare and encompass a heterogeneous group of entities, including pancreatoblastoma, pancreatic neuroendocrine tumors, congenital pancreatic cysts, and inflammatory pseudocysts. Solid pseudopapillary neoplasm (SPN) of the pancreas, also known as Frantz’s tumor, represents a distinct clinicopathologic entity within this spectrum and is a rare entity in pediatric age, accounting for an annual incidence rate of 0.2 per 10,00,000 children [1,2]. Despite their rarity, SPNs are the most common pancreatic neoplasm in children [3,4]. This tumor is predominantly diagnosed in females, with a female-to-male ratio between 8-20:1 [5,6]. SPNs often present insidiously, with nonspecific symptoms such as abdominal pain, distension, or abdominal mass. Sometimes it can be found as an incidentaloma [7]. However, due to their indolent nature, they can remain asymptomatic until reaching a considerable size or causing compression of adjacent structures. These tumors are slow-growing and usually do not cause pancreatic duct dilation [8].

Diagnostic evaluation of SPNs typically involves a multimodal approach, incorporating radiological imaging, histopathological examination, and immunohistochemical analysis. Ultrasound, computed tomography (CT), and magnetic resonance imaging (MRI) play a pivotal role in characterizing the lesion and planning the surgical procedure [9]. Definitive diagnosis relies on histopathology and immunohistochemistry, with SPNs typically expressing nuclear beta (β)-catenin and showing positivity for CD56, cyclin D1, and sometimes synaptophysin [10,11]. Although SPNs typically appear as well-encapsulated solid or mixed solid-cystic masses, atypical presentations have been described. In rare instances, SPNs may exhibit inflammatory features or mimic pancreatic pseudocysts on imaging, potentially leading to diagnostic delay or misclassification as benign inflammatory disease. Awareness of these unusual presentations is essential to ensure timely diagnosis and appropriate surgical management.

Given their low malignant potential, complete surgical resection remains the cornerstone of treatment for localized SPNs, offering favorable long-term outcomes [12,13]. On the other hand, local recurrence and distant metastasis have been reported following incomplete resection. We present a case of SPN diagnosed in an 11-year-old girl with an inflammatory and pseudocyst-like presentation, highlighting an important diagnostic pitfall and emphasizing key diagnostic and therapeutic considerations in pediatric practice.

## Case presentation

We report the case of an 11-year-old girl who presented to the emergency department with severe abdominal pain that had progressively worsened over a 24-hour period. She also exhibited fever (maximum temperature of 40°C), vomiting, and anorexia. The patient denied experiencing diarrhea or other associated symptoms. There was no reported history of trauma or pancreatitis, and no known epidemiological context for the illness.

On physical examination, she was hemodynamically stable. The patient was anicteric, with no signs of acholic stools or choluria. Her abdomen was soft and presented with normoactive bowel sounds, but she complained about mild tenderness and guarding in the right iliac fossa. Blumberg's sign was equivocal. No palpable organomegaly or lymphadenopathy was detected. The remaining examination was unremarkable.

Laboratory evaluation showed mild leukocytosis with neutrophilia and elevated C-reactive protein and erythrocyte sedimentation rate (Table 1). There were no analytical criteria suggestive of pancreatitis, cholestasis, or hepatocellular injury. Tumor markers were within normal limits. Serology for *Echinococcus granulosus* was negative. Bacteria, viruses, and parasites, including *Giardia lamblia*, were not detected in the stool sample. Urinalysis showed no active sediment, and all other biochemical parameters were within normal limits. Three gastric aspirate samples tested negative for *Mycobacterium tuberculosis*. An additional antigen-based nasal swab tested negative for viruses. Blood cultures were negative.

**Table 1:** List of complementary diagnostic tests

Test	Result	Reference range
Complete blood count		
Hemoglobin	11.7	11.5 – 15.5 g/dL
Leukocytes	14360	4500 – 13500 /uL
Neutrophils	11000	1800 – 8000 /uL
Lymphocytes	2000	1500 – 6500 /uL
Platelets	236000	> 150000 /uL
Erythrocyte sedimentation rate	95	1 – 20 mm/h
Blood chemistry		
Glucose	95	70 – 110 mg/dL
Urea	10	10.7 – 38.5 mg/dL
Creatinine	0.46	0.5 – 1.1 mg/dL
Sodium	137	135 – 145 mmol/L
Potassium	3.6	3.5 – 5.1 mmol/L
Chloride	95	98 – 107 mmol/L
Alkaline phosphatase (ALP)	77	141 – 460 U/L
Gamma-glutamyl transferase (GGT)	16	7 – 21 U/L
Aspartate aminotransferase (AST)	39	< 50 U/L
Alanine transaminase (ALT)	10	7 – 40 U/L
Amylase	51	25 – 101 U/L
Lipase	40.9	4 – 39 U/L
Albumin	3.4	3.5 – 5 g/dL
C-Reactive Protein (CRP)	35.7	< 5 mg/L
Coagulation profile		
International normalized ratio (INR)	1.1	0.8 – 1.2
Activated partial thromboplastin time (aPTT)	30.4	25 – 35 seconds
Prothrombin time (PT)	12.1	11 – 13.5 seconds
Urinalysis		
Chemical analysis	pH 7	4.5 – 8
	Specific gravity 1.012	1.003 – 1.030
	Negative (protein, glucose, ketones, bilirubin, urobilinogen, blood, nitrites, leukocyte esterase)	-
Serum tumor markers		
Carcinoembryonic antigen (CEA)	< 0.5	0.0 – 5.0 ng/mL
Carbohydrate antigen 19-9 (CA 19-9)	8.61	< 37 U/mL
Alpha-fetoprotein (AFP)	1.44	0 – 8 ng/mL
Microbiology		
Hemoculture	Negative	-
Stool culture and parasitological examination (including *Salmonella*, *Campylobacter*, *Shigella*, *Yersinia*, *Escherichia coli*, and *Giardia lamblia*)	Negative	-

Notably, the combination of elevated inflammatory markers with normal amylase and lipase levels represented an important diagnostic red flag, arguing against acute pancreatitis and prompting further investigation for alternative causes of acute abdomen.

**Figure 1:**
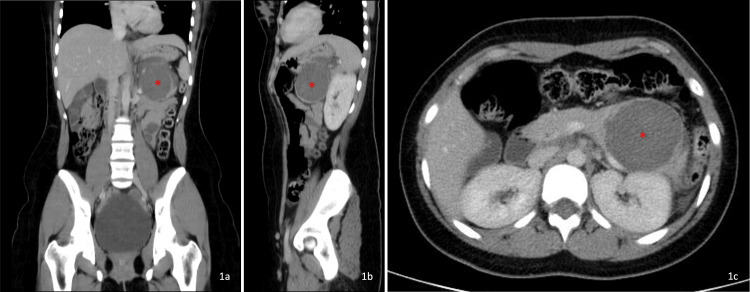
Abdominal and pelvic computed tomography (CT) with an abdominal window A well-defined cystic lesion was identified in the tail of the pancreas (red "*"), visualized on coronal (Figure 1a), sagittal (Figure 1b), and axial (Figure 1c) planes on contrast-enhanced CT in the venous phase. It demonstrated homogeneous low attenuation after contrast administration, without an apparent solid component (27 Hounsfield units); an unenhanced phase was not performed. The lesion measured approximately 68.1 × 56 mm in its largest dimensions. A small amount of free fluid was noted in the left paracolic gutter (Figure 1c). No other significant findings were identified.

**Figure 2:**
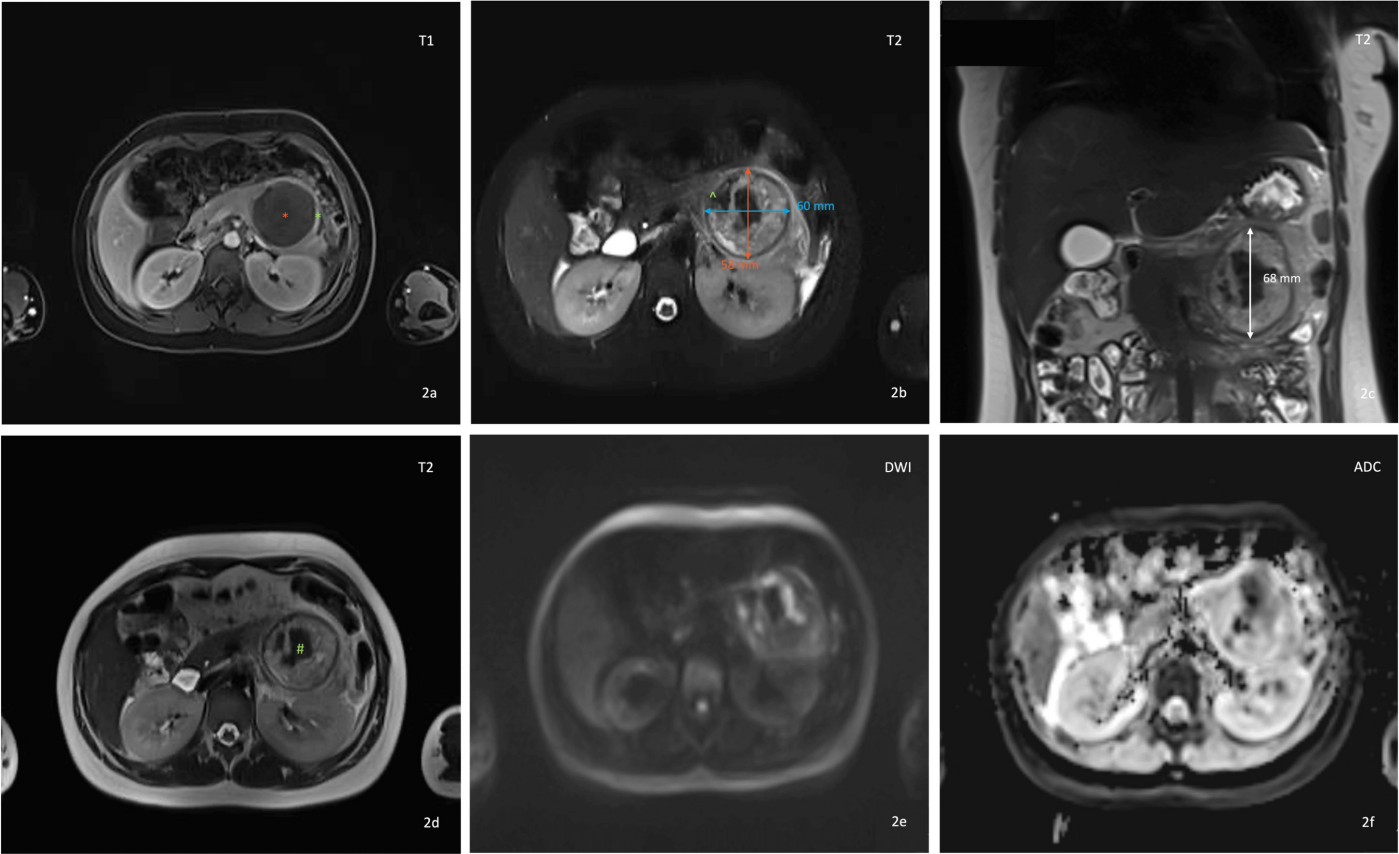
Abdominal magnetic resonance imaging (MRI) with cholangiographic study A large heterogeneous mass (red “*”) arising from the pancreatic tail was identified (Figure 2a), measuring approximately 68 × 60 × 58 mm on axial (Figure 2b) and coronal images (Figure 2c). Figure 2a (axial fat-suppressed T1-weighted post-contrast sequence) demonstrated mild peripheral enhancement (green “*”) after intravenous gadolinium administration. Figure 2b (axial fat-suppressed T2-weighted post-contrast sequence) demonstrated disruption of the wall/capsule (green “^”) at the anteromedial aspect, with posterior extension of the inflammatory process, predominantly at the posteroinferior aspect. This finding was associated with edematous infiltration of the adjacent tissue planes and a small fluid collection extending into the left anterior pararenal space and the left paracolic gutter. Figure 2d (axial T2-weighted sequence) showed that the lesion had a visible wall and displayed a heterogeneous T2 signal (green “#”), with areas of hypointensity, intermediate signal, and hyperintensity. Figure 2e and Figure 2f (axial diffusion-weighted magnetic resonance images (DWI) acquired with a b value of 1500 s/mm² and the corresponding apparent diffusion coefficient (ADC) map) showed no true diffusion restriction, as indicated by the absence of signal hyperintensity on DWI and the lack of corresponding signal reduction on the ADC map.

The initial abdominal and pelvic ultrasound was normal. Abdominal and pelvic CT (Figure 1), using an abdominal window, revealed a cystic lesion in the tail of the pancreas, measuring 68.1 x 56 millimeters (mm) in its largest dimensions. The lesion showed homogeneous low attenuation after contrast administration, without an apparent solid component. The remaining abdominal organs, including the gastrointestinal tract, were normal. A small amount of free fluid was present in the left paracolic gutter. For further clarification and assessment of solid components, an abdominal MRI was subsequently performed (Figure 2). Abdominal MRI with cholangiographic study showed a large heterogeneous mass arising from the pancreatic tail (Figure 2a), measuring 68 (L) x 60 (T) x 58 (AP) mm (Figure 2b and Figure 2c). Following intravenous gadolinium administration, there was mild peripheral enhancement (Figure 2a). The lesion had a visible wall and displayed a heterogeneous T2 signal, with areas of hypointensity, intermediate signal, and hyperintensity (Figure 2d). On the posteroinferior aspect, there was a disruption of the wall/capsule with extension of the inflammatory process posteriorly, associated with edematous infiltration of adjacent tissue planes and a small fluid collection extending into the left anterior pararenal space and the left paracolic gutter (Figure 2b). A small fluid collection was also noted in the right anterior pararenal space. The lesion showed no diffusion restriction and no significant internal enhancement (Figure 2e and Figure 2f). No signs of vascular invasion were observed. Bilateral pleural effusion was noted, more pronounced on the left side. No abnormalities were detected in the liver, gallbladder, biliary duct, spleen, adrenal glands, or kidneys. The aorta had a normal caliber. There was no evidence of lymphadenopathies or bowel wall thickening.

From a radiologic standpoint, the lesion demonstrated several features atypical for a pancreatic pseudocyst, including its large size (>60 mm), well-defined capsule, heterogeneous internal T2 signal, absence of antecedent pancreatitis or trauma, and lack of internal debris or hemorrhagic layering. The presence of mild peripheral enhancement, a visible capsule with focal disruption, and surrounding inflammatory infiltration suggested secondary inflammatory change of an underlying neoplasm rather than a primary inflammatory collection. Taken together, the absence of a history of pancreatitis or trauma, the acute inflammatory clinical presentation, normal pancreatic enzyme levels, the absence of imaging signs of pancreatitis, and the radiologic findings represented key red-flag features indicating an underlying pancreatic neoplasm with secondary inflammatory changes rather than a primary inflammatory or post-pancreatitis pseudocyst.

Given the acute inflammatory presentation, high-grade fever, markedly elevated inflammatory markers, and imaging evidence of perilesional inflammatory extension and small fluid collections, an initial conservative approach with broad-spectrum intravenous antibiotics was adopted to control the presumed secondary infection or sterile inflammatory reaction before definitive surgical intervention. The patient was admitted and initially treated with intravenous amoxicillin-clavulanate for six days. She became afebrile after three days of therapy. Subsequently, her antibiotic regimen was switched to piperacillin-tazobactam, which was administered for eight days.

Following multidisciplinary assessment, it was determined that elective surgical resection should be delayed for four to six weeks after resolution of the acute inflammatory process in order to reduce operative morbidity, facilitate safer dissection planes, and decrease the risk of postoperative complications. Given the risk of concomitant splenectomy during surgery, the patient received several vaccines two weeks prior to the procedure, including the quadrivalent meningococcal conjugate vaccine targeting serogroups A, C, W, and Y (MenACYW), the 23-valent pneumococcal polysaccharide vaccine, and the influenza vaccine. She had already completed the vaccination schedule for the *Haemophilus influenzae* type b conjugate vaccine (Hib), meningococcal group B vaccine (MenB), and 13-valent pneumococcal conjugate vaccine (PCV13).

She underwent a laparoscopic spleen-preserving distal pancreatectomy, with preservation of the splenic vessels (Kimura technique), which was uneventful. No evidence of tumor spread was observed intraoperatively. Histological evaluation showed a well-defined, non-capsulated neoplasm with extensive necrosis associated with histiocytes and cholesterol clefts. A peripheral rim of viable neoplastic tissue was observed, infiltrating the surrounding pancreatic tissue, composed of monotonous cell nests with clarified cytoplasm and vesicular nuclei containing clefts. Perineural invasion was observed, while lymphovascular invasion was not identified. Surgical resection margins were negative for invasive carcinoma. In immunohistochemistry, the neoplastic cells showed diffuse nuclear and cytoplasmic expression of β-catenin, as well as cyclin-D1 and CD56. Synaptophysin showed multifocal positivity, and CD10 weak expression. CD99 and chromogranin were negative. The Ki-67 proliferation index was low (<2%), consistent with the indolent behavior typically observed in SPNs. The morphology and immunohistochemistry were consistent with a diagnosis of SPN of the pancreas. Additionally, five regional lymph nodes were resected, all of which were negative for metastasis. Thus, the pathologic stage, according to the 8th edition of the American Joint Committee on Cancer (AJCC), was pT3N0M0 [14].

Postoperatively, the patient developed a biochemical pancreatic fistula, without clinical repercussions (grade A), with persistent serous drainage from the surgical site. The fistula was defined and graded according to the 2016 International Study Group of Pancreatic Surgery (ISGPS) criteria [15]. Management was conservative, consisting of maintenance of surgical drainage, close monitoring of output and biochemistry, and temporary hypocoagulation therapy to prevent thrombosis due to the presence of intra-abdominal inflammation and altered perfusion. No signs of secondary infection or intra-abdominal collections were identified. The patient remained clinically stable, tolerated reintroduction of diet, and showed progressive reduction in drain output. She was discharged on postoperative day 8, and the abdominal drain was maintained for approximately three weeks until resolution of the fistula. She remains under follow-up in pediatric surgery consultation, with no other significant intercurrent events.

## Discussion

SPN of the pancreas remains a rare entity, though it is the most common pancreatic neoplasm in the pediatric population [3]. Its indolent clinical course allows lesions to grow and reach considerable size before diagnosis. In our case, the adolescent presented with symptoms attributable to the tumor, which is not the most commonly reported presentation in the literature [7].

SPN primarily affects adolescent females, possibly due to hormonal influences [5]. Our patient reflects this demographic pattern, being an 11-year-old girl with a lesion in the pancreatic tail, a site more commonly involved in pediatric SPNs compared to adults [4].

Radiologic evaluation, particularly CT and MRI, plays a central role in lesion detection and surgical planning [9]. Characteristically, SPNs appear as well-encapsulated heterogeneous masses with solid and cystic components and peripheral enhancement. However, variations in enhancement and morphology can hinder the diagnosis. MRI often reveals hemorrhagic and heterogeneous signals with capsular enhancement [6]. In this patient, the imaging raised a differential diagnosis between SPN and a ruptured pancreatic pseudocyst. The presence of surrounding inflammation, small fluid collections in the retroperitoneum, and the lesion’s cystic nature raised suspicion of pseudocyst rupture. Notably, SPNs are usually associated with more prominent contrast enhancement than was observed here. However, no history of abdominal trauma, pancreatitis, or pancreatic ductal abnormalities was identified, factors that typically support a pseudocyst diagnosis [7,8]. Extensive necrosis, wall disruption, and associated inflammatory changes have been documented in large SPNs and may mimic cystic inflammatory lesions [10,11].

Histologically, SPNs exhibit pseudopapillary architecture with uniform cells and characteristic immunohistochemical staining, diffuse nuclear β-catenin expression, positivity for CD56, CD10, and cyclin D1, and negativity for chromogranin [10,11]. These findings were present in our case, confirming the diagnosis. In comparison with reported pediatric SPN series, this case shares several typical features, including female sex, large tumor size at diagnosis, and indolent histopathologic characteristics with low Ki-67 proliferation index. However, the acute inflammatory presentation with fever, marked elevation of inflammatory markers, capsular disruption, and extensive intratumoral necrosis is distinctly uncommon and has been only rarely described in pediatric patients. Most pediatric SPNs are incidentally discovered or present with nonspecific abdominal pain rather than acute abdomen. This atypical presentation contributed to the initial radiologic impression of a pseudocyst and highlights the importance of maintaining a high index of suspicion for SPN in children presenting with large pancreatic cystic lesions, even in the absence of classic solid components.

Complete surgical resection with negative margins is the gold standard for treatment, with over 95% five-year survival [12]. Spleen-preserving distal pancreatectomy is preferred in children when anatomically feasible, as performed in our case, because it reduces the risk of infections caused by encapsulated bacteria and postoperative complications [13]. Incomplete resection increases the risk of recurrence or metastasis, underscoring the importance of complete tumor removal [12]. A pancreatic fistula is one of the most common postoperative complications following pancreatic surgery in both adult and pediatric populations. Although SPN is considered low-grade, surgery involving the pancreatic parenchyma carries a measurable risk of fistula formation due to enzymatic leakage from transected ducts. In pediatric SPN series, the incidence of pancreatic fistula has been reported between 5% and 15% [7,8,9]. Most are grade A or B according to the ISGPS criteria and resolve with conservative management, including nil per os (NPO), octreotide, and drain care [15].

While prognostic markers such as Ki-67 index >4% and lymphovascular invasion may indicate aggressive behavior, most pediatric cases have a benign course [11]. Long-term surveillance remains essential due to the potential for late recurrence [11].

This case highlights the importance of considering SPN in the differential diagnosis of atypical cystic-solid pancreatic lesions in children, even in the absence of classic features or significant enhancement. Awareness of its varied presentations is essential to avoid misdiagnosis and ensure timely curative surgery.

## Conclusions

SPN of the pancreas, although rare in the pediatric population, should be considered in the differential diagnosis of pancreatic masses in children and adolescents, particularly in female patients. This case illustrates an atypical clinical presentation associated with acute inflammatory features and pseudocyst-like imaging findings that mimicked an inflammatory pancreatic lesion, thereby generating significant diagnostic uncertainty and highlighting the diagnostic challenges posed by this entity.

A multimodal diagnostic approach, combining advanced imaging techniques with histopathological and immunohistochemical analysis, was essential for establishing the definitive diagnosis. Complete surgical resection remains the treatment of choice and was safely achieved in this patient through a minimally invasive, spleen-preserving distal pancreatectomy, resulting in an excellent clinical outcome. This report underscores the importance of early recognition of solid pseudopapillary neoplasm and appropriate surgical management, and emphasizes the need for long-term follow-up given the potential, albeit low, risk of late recurrence.
